# Non-immune-mediated versus immune-mediated type 1 diabetes: diagnosis and long-term differences—retrospective analysis

**DOI:** 10.1186/s13098-020-00563-x

**Published:** 2020-07-06

**Authors:** Diana Catarino, Diana Silva, Joana Guiomar, Cristina Ribeiro, Luísa Ruas, Luís Cardoso, Isabel Paiva

**Affiliations:** 1 Endocrinology, Diabetes and Metabolism Department, Centro Hospitalar e Universitário de Coimbra EPE, Praceta Prof. Mota Pinto, 3000-075 Coimbra, Portugal; 2 Medicine Faculty of Coimbra University, Coimbra, Portugal

**Keywords:** Non-immune-mediated diabetes mellitus, Immune-mediated diabetes mellitus, Dyslipidemia, Total daily insulin dose, Microvascular complications, macrovascular complications

## Abstract

**Background:**

The American Diabetes Association proposed two subcategories for type 1 diabetes mellitus: type 1A or immune-mediated diabetes (IDM) and type 1B or idiopathic diabetes. The absence of β-cell autoimmune markers, permanent insulinopenia and prone to ketoacidosis define the second category, whose pathogenesis remains unclear. Only a minority of patients fall into this category, also designated non-immune-mediated (NIDM), which is considered by several authors similar to type 2 diabetes. The aim of this study is to evaluate differences at the diagnosis and 10 years later of two categories.

**Methods:**

Retrospective cohort study of patients with β-cell autoimmune markers performed at diagnosis and undetectable c-peptide. Were excluded patients with suspicion of another specific type of diabetes. We obtained two groups: IDM (≥ 1 positive antibody) and NIDM (negative antibodies). Age, family history, anthropometry, duration of symptoms, clinical presentation, blood glucose at admission, A1C, lipid profile, arterial hypertension, total diary insulin dose (TDID), microvascular and macrovascular complications were evaluated. Results were considered statistically significant with p < 0.05.

**Results:**

37 patients, 29 with IDM and 8 patients with NIDM. The age of diagnosis of IDM group (23 years) was significantly different (p = 0.004) from the NIDM group (38.1). The body mass index (BMI) at the diagnosis did not differ significantly (p = 0.435). The duration of symptoms was longer in the NIDM (p = 0.003). The disease presentation (p = 0.744), blood glucose (p = 0.482) and HbA1c (p = 0.794) at admission and TDID at discharge (p = 0.301) did not differ significantly. Total and LDL cholesterol levels were higher in NIDM group but did not differ significantly (p = 0.585 and p = 0.579, respectively). After 10 years BMI did not differ between groups (p = 0.079). Patients with IDM showed a significantly higher HbA1c (p = 0.008) and TDID (p = 0.017). Relative to the lipid profile, there was no significant difference, however the LDL cholesterol and triglycerides were higher on the NIDM group, as the percentage of hypertension. Microvascular complications were higher in the IDM group, but no significant difference was found.

**Conclusion:**

Patients with IDM had a poor metabolic control and higher insulin requirement. Patients with NIDM were older and showed higher cardiovascular risk, resembling a clinical phenotype of type 2 diabetes.

## Background

In 1997, the American Diabetes Association proposed two subcategories for type 1 diabetes mellitus: type 1A or immune-mediated diabetes and type 1B or idiopathic diabetes [1, 2].

The immune-mediated diabetes (IDM) results from a cellular autoimmune destruction of the β-cells of the pancreas, mediated by T-cells [3, 4]. Markers of the immune destruction of the β-cell include islet cell autoantibodies, insulin autoantibodies, GAD (GAD65) autoantibodies and tyrosine phosphatase (IA2) autoantibodies. There is little or no insulin secretion, manifested by low or undetectable levels of plasma C-peptide [4], and exogenous insulin is necessary to preserve life. Insulin resistance does not play a major role in its pathogenesis [3]. The disease has strong HLA (human leukocyte antigen) haplotypes associations, with linkage to the DQA and DQB genes. The IDM commonly occurs in childhood and adolescence, but it can occur at any age and patients are rarely obese at the diagnosis [4].

The idiopathic diabetes is characterized by absence of β-cell autoimmune markers, with permanent insulinopenia and prone to ketoacidosis [1, 2, 4]. The authors of this paper evoked this type of diabetes by non-immune-mediated diabetes mellitus (NIDM).

Only a minority of patients with type 1 diabetes mellitus fall into this subcategory, however it is being recognized as an important clinical entity [5].

NIDM has been mostly described in African-American and Asian patients, even though it has also been described in native Americans and in European Mediterranean individuals [1–3].

Although patients with NIDM have generally an onset similar to that of patients with IDM, some differences are frequently found.

NIDM is characterized by acute onset of severe hyperglycemia with ketoacidosis, requiring hospital admission and treatment with insulin and fluid and electrolyte replacement [5]. Insulin therapy is generally necessary for a period going from 6 to 18 months, with subsequent good control of disease just with oral agents and diet [2]. Recurrent ketoacidosis is unusual [5].

NIDM shows a different phenotype, are more often male, middle aged, overweight, or modestly obese (obesity class I). They have a family history of type 2 diabetes [2, 3, 5, 6]. Due to the presence of some metabolic features of type 2 diabetes, the NIDM has also been referred in the literature as atypical diabetes, type 1.5 diabetes, Flatbush diabetes and ketosis-prone diabetes [5, 7–9].

Its pathogenesis is unknown, and the information about this is scare, but is likely related to insulin resistance and transient β-cell dysfunction, perhaps due to glucotoxicity and lipotoxicity mechanisms [2, 3, 10]. HLA-related genes are not believed to be involved in its pathogenesis, even though mutations in different genes from HLA have been reported, suggesting that NIDM may have a specific genetic background.

Recently, at the Classification of diabetes mellitus 2019 of the World Health Organization, the NIDM was reclassified as ketosis-prone type 2 diabetes [11].

## Methods

This study was approved by the local ethics review boards (Coimbra Hospital and University Center). All Patients signed an informed consent for the scientific use of their data.

Retrospective cohort study, from January 2003 to December 2008, based on clinical records of patients with low C-peptide (< 0.5 ng/mL) and in which diabetes mellitus-related autoimmune markers (anti GAD-65, anti-islets, anti-insulin, anti IA2) were measured. Only patients whose assays were performed at the time of diagnosis of diabetes mellitus were considered to ensure the inclusion of patients with type 1 DM. Of these, we obtained two groups: one with positive autoimmunity—IDM group (≥ 1 positive antibody) and another with negative autoimmunity—NIDM group. Differences between groups at diagnosis were evaluated with regard to age of diagnosis, family history, anthropometry, duration of symptoms, clinical presentation of disease, plasma glucose at hospital admission, HbA1c, lipid profile, arterial hypertension and total daily insulin dose (TDID).

The authors also analyzed differences between the groups at long term follow-up-ten years—with regard to anthropometry, HbA1c, lipid profile, arterial hypertension, TDID, microvascular and macrovascular complications.

C-peptide measurement was performed after correction of ketosis or ketoacidosis and stabilization of plasma glucose levels. The lipid profile was obtained in the first medical evaluation after discharge.

In this study, all patients included were treated with conventional basal-bolus therapy.

All patients were Caucasian.

Categorical variables are presented as frequencies and percentages, and continuous variables as means and standard deviations, or medians and interquartile ranges for variables with skewed distributions. Normal distribution was checked using skewness and kurtosis. All reported p values are two-tailed, with a p value of 0.05 indicating statistical significance.

The differences between groups were detected by the Student’s t test for continuous variables with normal distribution, by the Mann–Whitney test and Wilcoxon test for continuous variables without normal distribution and by the χ^2^ test for categorical variables.

Analyses were performed with the use of SPSS v.25.

## Results

### Differences at diagnosis

At diagnosis, 29 patients (78.4%) had positive autoimmune markers and 8 had negative autoimmune markers. In the group with positive autoimmunity, 15 patients were female (48.3%), while in the group with negative autoimmunity they were all male.

In the IDM group, the median age of patients at diagnosis was 23.0 (9) years, and in the NIDM group the mean age at diagnosis was 38.1 ± 12.8 years, with a statistically significant difference (p = 0.004).

BMI at diagnosis did not differ significantly (p = 0.435) between the two groups (20.97 kg/m^2^ in IDM vs 20.37 kg/m^2^ in NIDM). There was no statistically significant association between groups and family history of type 1 DM (p = 0.999) or type 2 DM (p = 0.999).

Symptoms duration in both patient groups was statistically different (p = 0.003), with a duration of 21.8 ± 8.8 days in the IDM group vs 45.0 (60) days in the NIDM group, but there was no statistically significant association between the groups and the clinical presentation of the disease (p = 0.744).

Plasma glucose at hospital admission was not statistically different (25.47 mmol/L in the IDM group vs 23.92 mmol/L in the NIDM group) (p = 0.482), such as HbA1c at diagnosis (11.3% vs 11.8%, respectively) (p = 0.794).

With regard to lipid profile, total-cholesterol, LDL-C, HDL-C and triglycerides levels, they did not differ significantly between the two groups (p = 0.585, p = 0.579, p = 0.833 and p = 0.555, respectively), although total-cholesterol and LDL-C levels were higher in the NIDM group. The percentage of patients with dyslipidemia was higher in the NIDM group (25% vs 24.1%), however the difference was not statistically significant (p = 0.999). With regard to arterial hypertension, there was no significant difference between the groups (p = 0.999).

The TDID at discharge was not statistically different (46.4 units vs. 40.1 units) (p = 0.301) (Table 1).

**Table 1:** Clinical and metabolic parameters in patients with IDM and NIDM at diagnosis

	IDM group (n = 29; 78.4%) Mean ± SD Median (IQR)	NIDM group (n = 8; 21.6%) Mean ± SD Median (IQR)	p value
Clinical parameters			
Age (years)	23.0 (9)	38.1 ± 12.8	0.004*
BMI (Kg/m^2^)	20.97 (3.5)	20.37 ± 2.7	0.435
Family history type 1/2 DM (%)	17.2/37.9	12.5/37.5	0.999
Symptoms duration (days)	21.8 ± 8.8	45.0 (60)	0.003*
Presentation of the disease			0.744
Arterial hypertension (%)	6.9	0	0.999
Metabolic parameters			
Plasma glucose at admission (mmol/L)	25.47 (8.16)	23.92 (8.66)	0.482
HbA1c (%)	11.3 ± 2.2	11.8 (2.5)	0.794
TDID (U)	46.4 ± 15.3	40.1 ± 12.3	0.301
Dyslipidemia (%)	24.1	25.0	0.999
Total-C (mmol/L)	8.77 (2.61)	9.05 (2.72)	0.585
LDL-C (mmol/L)	5.57 ± 1.44	5.90 ± 1.47	0.579
HDL-C (mmol/L)	2.75 ± 0.83	2.80 (0.50)	0.833
Triglycerides (mmol/L)	4.73 (1.37)	3.72 (2.05)	0.555

SD: standard deviation; IQR: interquartile range

p* < 0.05: statistically significant difference

Presentation of the disease: diabetic ketoacidosis; hyperglycemic hyperosmolar syndrome; polyuric polydipsic syndrome; diabetic ketoacidosis with hyperosmolarity; seizures

### Differences at 10 years of follow-up

At ten years evaluation, BMI was not statistically different (p = 0.079) between the two groups (25.14 kg/m^2^ in IDM group vs 22.58 kg/m^2^ in NIDM).

Relative to HbA1c there was statistically significant difference between the groups (p = 0.008), with 8.7% for IDM group and 7.4% for NIDM group.

The insulin requirement was also statistically different. The TDID of the IDM group was 52.35 units and the NIDM group was 33.5 units (p = 0.017).

The percentage of patients with dyslipidemia was higher in the NIDM group (62.5% vs 44.8%), however the difference was not statistically significant (p = 0.999).

With regard to lipid profile, total-cholesterol, LDL-C, HDL-C and triglycerides levels there was no statistically significant difference between the two groups (p = 0.728, p = 0.571, p = 0.338, p = 0.648, respectively), however the LDL-C and triglycerides levels were higher in the NIDM group.

The percentage of patients with hypertension was higher in the NIDM group (25% vs 17.2%), although there was no significant difference between the groups (p = 0.999).

With regard to microvascular complications, there was no statistically significant difference at the percentage of retinopathy, neuropathy and nephropathy between the two groups (p = 0.550, p = 0.550, p = 0.550, respectively) but the percentage was higher in the IDM group. At 10 years follow-up, the NIDM group had not microvascular complications.

There was no significant difference on the macrovascular disease too (Table 2).

**Table 2:** Clinical and metabolic parameters in patients with IDM and NIDM at 10 years of follow-up

	IDM group (n = 29; 78.4%) Mean ± SD Median (IQR)	NIDM group (n = 8; 21.6%) Mean ± SD Median (IQR)	p value
Clinical parameters			
BMI (Kg/m^2^)	25.1 (4.2)	22.6 ± 3.5	0.079
Arterial hypertension (%)	17.2	25.0	0.999
Metabolic parameters			
HbA1c (%)	8.7 (1.7)	7.4 (1.0)	0.008*
TDID (U)	52.4 ± 16.7	33.5 (12)	0.017*
Dyslipidemia (%)	44.8	62.5	0.999
Total-C (mmol/L)	185.7 ± 43.2	184 (112)	0.728
LDL-C (mmol/L)	108.5(51)	112 (89)	0.571
HDL-C (mmol/L)	58.1 ± 13.7	58.4 ± 13.0	0.338
Triglycerides (mmol/L)	79(51)	83 (169)	0.648
Long term complications			
Microvascular complications			
Retinopathy	13.8	0	0.550
Neuropathy	10.3	0	0.550
Nephropathy	10.3	0	0.550
Macrovascular complications			
Coronary disease	0	12.5	0.250
Cerebrovascular disease	3.4	0	0.999
Peripheral arterial disease	0	0	NA

SD: standard deviation; IQR: interquartile range; NA: not applicable

p* < 0.05: statistically significant difference

We found a reduction in HbA1C at 10 years of follow-up in both groups [2.4 ± 2.6% in the IDM group; 3.9 (4.0)  % in the NIDM group]. The HbA1C reduction was higher in the NIDM group, although it did not differ significantly (p = 0.109). With regard to the variation of the TDID, the IDM group had a higher need for insulin at 10 years compared to the diagnosis (52.4 ± 16.7U at 10 years versus 46.4 ± 15.3U at diagnosis), unlike the NIDM group [33, 5 (12) U at 10 years versus 40.1 ± 12.3 U at diagnosis] (Table 3)

**Table 3:** Over-time variation of HbA1c between IDM and NIDM groups

	IDM group (n = 29; 78.4%) Mean ± SD Median (IQR)	NIDM group (n = 8; 21.6%) Mean ± SD Median (IQR)	p
HbA1c (%)	2.4 ± 2.6	3.9 ± 4.0	0.109

SD: standard deviation; IQR: interquartile range

p* < 0.05: statistically significant difference

## Discussion and conclusions

Our study suggests that the NIDM may be detected among subjects of Caucasian ethnicity and in spite of initial clinical presentation compatible with IDM, they differ at diagnosis in terms of autoimmune markers, sex, age of patients and symptoms duration.

All patients in the NIDM group included in our study were men, reporting a male predominance consistent with other studies [1–3, 12] So far, the cause of this male predominance is unknown, however it is thought that hormonal factors may be involved.

In the IDM group, the median age of patients at diagnosis was higher, in agreement with other studies [1, 7, 13].

Symptoms duration in both patient groups was statistically different, with a longer duration in the NIDM group, unlike other studies, in which there were no differences between the two groups [1].

BMI at diagnosis did not differ significantly between the two groups. In most previous studies, patients in the NIDM group have a higher BMI with visceral obesity, resembling patients with type 2 diabetes [1, 2].

There was no statistically significant association in the clinical presentation of the disease, as in previous studies [3, 7, 12], making sometimes difficult to differentiate it with the IDM group.

HbA1c at diagnosis and the TDID at discharge were not statistically different between the two groups, which is in agreement with other works [1, 2]. Total-cholesterol and LDL-C levels were higher in the NIDM group, as previously reported in other studies, where patients from this group have a more atherogenic lipid profile, as patients with type 2 diabetes [2].

At a long-term evaluation, our study shows that patients with IDM had a poor metabolic control, with higher HbA1c and higher insulin requirement, consistent with previous studies [2]. On the other hand, in the NIDM group there was a higher HbA1c reduction with lower insulin requirement.

In fact, other studies reported a severe insulin secretory deficiency only during the acute ketotic phase in patients with NIDM with a clinical remission phase correlated to an insulin secretion recovery [2].

Patients with NIDM showed a lower tendency to microvascular complications, like type 2 diabetes. Microvascular complications were more frequent in the IDM group.

Patients with NIDM showed, as at the diagnosis, a typical atherogenic lipid profile, characterized at 10 years by high LDL-cholesterol and triglycerides levels. This group also showed a higher proportion of arterial hypertension patients. So, the authors concluded that NIDM is associated with higher cardiovascular risk than IDM since the diagnosis.

There are some limitations of this study, that should be mentioned, such as the sample size that was conditioned by the retrospective nature of the study. The authors were limited to patients whose assays were made at diagnosis to avoid misdiagnosis.

In conclusion, recognition of the NIDM category is critical in clinical practice because it may modify the therapeutic approach of these patients, in the mid and long term. This entity was initially diagnosed in Asian and African American populations, however, individuals from other ethnic groups, namely Caucasian, have been increasingly identified. The initial clinical presentation is similar to the IDM group, requiring intensive initial insulin therapy and fluid and electrolyte replacement. However, behind the glucotoxicity and lipotoxicity phase, there is functional recovery of the β cell after several weeks, which allows in most patients an approach with diet alone or diet plus oral medications.

The pathophysiological mechanisms leading to the acute onset of severe hyperglycemia, with or without ketosis and ketoacidosis in susceptible patients are unknown; hence further investigation in this area is needed.

The recognition of NIDM patients is also crucial because they are exposed to a higher cardiovascular risk needing adequated treatment to reduce the long-term cardiovascular complications.

## Data Availability

All data generated or analysed during this study are included in this published article.

## Abbreviations

DM: Diabetes mellitus
NIDM: Non-immune-mediated diabetes mellitus
IDM: Immune-mediated diabetes mellitus
TDID: Total daily insulin dose
HLA: Human leukocyte antigen
GAD: Glutamic acid decarboxylase
